# Trajectories of medication use after whiplash injury: high rates of persistent opioid and sedative use

**DOI:** 10.1097/PR9.0000000000001513

**Published:** 2026-11-12

**Authors:** Harpa Ragnarsdóttir, Lárus Steinþór Guðmundsson, Freyja Jónsdóttir, Hjalti Már Björnsson

**Affiliations:** a Research Centre of Movement Science, Department of Physiotherapy, University of Iceland, Reykjavík, Iceland; b Elja Physiotherapy, Reykjavík, Iceland; c Faculty of Pharmaceutical Sciences, University of Iceland, Reykjavik, Iceland; d Pharmacy Services, Landspitali-The National University Hospital of Iceland, Reykjavík, Iceland; e Faculty of Medicine, University of Iceland, Reykjavík, Iceland; f Emergency Department, Landspitali-The National University Hospital of Iceland, Reykjavík, Iceland

## Abstract

**Introduction:**

Medications are commonly used to manage symptoms after Whiplash-Associated Disorders, although evidence supporting longterm use remains limited, particularly for opioids and sedative/hypnotic medications. **Objective:** We aimed to quantify longitudinal changes in medication redemption before and after motor vehicle collision (MVC)–related whiplash injury and to examine whether early postinjury pain and disability are associated with subsequent medication trajectories.

**Methods:**

In this single-center nested prospective cohort study, 167 adults with MVC-related whiplash injury were included. Subacute measures of pain intensity (Visual Analog Scale) and disability (Neck Disability Index) were linked to nationwide prescription registry data covering 12 months before and after injury. Three medication categories were examined: nonopioid analgesics, opioids, and sedatives/hypnotics. Outcomes included dispensed defined daily doses and user trajectories (nonuse, prior use, new use, and new persistent use). Mixed-effects models and logistic regression analyses were applied.

**Results:**

The proportion of individuals redeeming analgesics increased from 19% to 35% and opioids from 34% to 51%, while sedatives/hypnotics showed a modest, nonsignificant increase (13%–17%). Among new users, 26% of opioid users and 43% of sedative/hypnotic users progressed to persistent use. Higher disability and pain were associated with greater medication use. Women had higher odds of initiating and persisting in opioid use.

**Conclusion:**

Medication redemption increases after MVC-related whiplash injury, with substantial progression to persistent use among new opioid and sedative/hypnotic users. Early symptom burden and patient characteristics may help identify individuals at risk, supporting guideline-concordant prescribing, careful monitoring of prolonged medication use, and nonpharmacological management where appropriate.

## Abstract 2

Medication use increased after whiplash injury, many progressed to persistent opioid and sedative use. Higher pain, disability, and female sex predicted greater and prolonged use.

## 1. Introduction

Whiplash Associated Disorders (WAD) commonly arise after motor vehicle collisions (MVC)^7^ and represent a substantial clinical and societal burden.^19,26,32^ Although many individuals recover, a considerable proportion develop persistent pain and disability,^16,21^ contributing to long-term healthcare utilization and reduced work capacity.^19,26^ Despite extensive research, effective management strategies for subacute and chronic WAD remain limited, and treatment responses are often suboptimal.^42,43,45^

Pharmacological management is frequently used to address symptoms during the acute phase of WAD.^30,40^ Commonly prescribed medications include nonopioid analgesics, opioids, and sedative/hypnotics.^30^ While clinical guidelines generally recommend cautious, short-term use of simple analgesics while discouraging prolonged use of opioids and other centrally acting medications,^40^ opioid use beyond the acute phase remains common in clinical practice.^13,30,36^ Evidence supporting the long-term benefit of these treatments is limited,^9,15^ and opioid use has not been shown to improve short-term pain outcomes compared with nonopioid analgesics, while being associated with a greater likelihood of continued use.^3^ In contrast, real-world prescribing patterns suggest that these recommendations are not consistently followed, and opioid prescribing remains prevalent.^36^ Notably, opioid prescribing rates have been reported to be comparable with referrals for physiotherapy, although clinical guidelines generally recommend exercise-based and other nonpharmacological interventions as part of WAD management, despite the evidence base remaining modest.^35,37,40,41^

Emerging evidence indicates that early exposure to opioids and other medications after injury may increase the risk of persistent use, even among individuals without prior use.^17,20,49^ This highlights a potential transition from short-term symptom management to long-term pharmacological reliance. Persistent use of opioids and sedative/hypnotics has been associated with adverse outcomes, including dependency and increased morbidity.^12^ Despite this, the integration of preinjury medication patterns with early clinical characteristics in longitudinal studies remains limited.

Understanding longitudinal patterns of medication use, before and after injury, may provide important insights into how pharmacological trajectories develop over time. In particular, examining both the amount of medication use and distinct user trajectories (eg, nonuse, prior use, new use, and new persistent use) may help identify subgroups at risk of prolonged pharmacological exposure. Furthermore, linking these trajectories to early clinical characteristics, such as pain intensity and disability, as well as demographic factors, may improve the identification of individuals at increased risk of sustained medication use. Therefore, the aim of this study was to quantify changes in redeemed medications before and after motor vehicle collision–related whiplash injury across 3 medication categories: nonopioid analgesics, opioids, and sedative/hypnotics. In addition, we aimed to examine patterns of medication use and to determine whether early postinjury pain, disability, and patient characteristics were associated with subsequent medication use trajectories. We hypothesized that medication use would increase after injury and that higher pain and disability, female sex, and other patient characteristics would be associated with a greater likelihood of new and persistent use.

## 2. Material and methods

The study was a single-center nested prospective cohort study examining changes in medication usage in relation to a whiplash injury to the neck after an MVC. The study reporting adheres to the STROBE guidelines for reporting of observational studies in epidemiology.^10^

### 2.1. Study population

The study population consisted of 167 participants who sustained a whiplash injury to the neck after an MVC during the study period from May 2022 until August 2023 and sought medical treatment at the Emergency Department at Landspitali University Hospital or at any of the 19 Health Care Centers of the Capital Region. Inclusion criteria were receiving, upon arrival, an International Classification of Primary Care code of “traffic accident” and an International Classification of Disease, 10th revision, S13.4 (sprain and strain of cervical spine), and 18 years and older. Information on additional traumatic injuries was not systematically collected and was therefore not included in the present analyses. Participants were excluded if they were unable to read/write Icelandic. Sample size was determined by recruitment feasibility within the parent trial; no formal power calculation for medication outcomes. Approval for the study was obtained from the National Bioethics Committee (Iceland VSN-21-093) and the protocol was registered (NCT05319808) and published before the end of recruitment (“Rehabilitation for Whiplash Associated Disorders” at clinicaltrials.gov, nr. NCT05319808. Patient enrollment started May 27, 2022).^34^ All participants signed an informed consent form. This present observational study was nested within a larger treatment study.

### 2.2. Clinical and medication data

The clinical data were obtained using questionnaires administered by the primary investigator during the subacute (1–3 months) phase of whiplash injury. Information on redeemed medication for 3 medication categories (over-the-counter [OTC] analgesics, opioids, and sedatives/hypnotics; Table 1) 12 months before and after the accident were retrieved from the Prescription Medicines Registry at the Directorate of Health database. This national electronic database contains real-time records of all outpatient redeemed drug prescriptions in Iceland. The database includes both regular and as-needed medications, but does not include over-the-counter, topical, or herbal medications.

**Table 1:** Classification of 3 medication categories by ATC* codes.

1: OTC† analgesics	2: Opioid analgesics	3: Sedatives and hypnotics‡
M01AB05: Diclofenac	N02AA01: Morphine	N05BA01: Diazepam
M01AE01: Ibuprofen	N02AA03: Hydromorphone	N05BA02: Chlordiazepoxide
M01AE02: Naproxen	N02AA05: Oxycodone	N05BA04: Oxazepam
N02BE01: Paracetamol	N02AA55: Oxycodone and naloxone	N05BA06: Lorazepam
N02BA51: Acetylsalicylic acid and caffeine	N02AB03: Fentanyl	N05BA08: Bromazepam
	N02AG02: Ketobemidone	N05BA09: Clobazam
	N07BC01: Buprenorphine	N05BA12: Alprazolam
	N07BC02: Methadone	N05CD01: Flurazepam
	N02AX02: Tramadol	N05CD02: Nitrazepam
	N02AJ06: Codeine and paracetamol	N05CD03: Flunitrazepam
		N05CD05: Triazolam
		N05CD08: Midazolam
		N05CF01: Zopiclone
		N05CF02: Zolpidem

* ATC: Anatomical Therapeutic Chemical Classification System.

† OTC: analgesics available over the counter in pharmacies.

‡ Benzodiazepines and benzodiazepine–related drugs.

### 2.3. Outcomes

The primary outcomes were (1) Defined Daily Doses (DDDs) of redeemed medication and (2) the proportion of individuals redeeming prescribed medication for each category. The secondary outcomes included scores on questionnaires and specific demographic and injury-related information. The Neck Disability Index (NDI),^46^ a 10-item self-report questionnaire, scored on a 0 to 5 Likert scale, expressed as a percentage (0%–100%), where a higher score indicates greater disability, with strong reliability and validity,^8,14,31^ was used to assess neck pain–related disability. Average neck pain intensity over the past week was assessed with the 100-mm Visual Analog Scale (VAS) (0 = no pain, 100 = worst imaginable pain), a widely used tool and validated in musculoskeletal populations.^8,33^ Demographic and injury-related information were collected using a structured questionnaire, which included questions about age, sex, and position in vehicle at the time of the accident (driver vs passenger), early symptom presentation (ie, whether participants reported acute symptoms such as head trauma, headache, or dizziness), education level, occupation, and salary range.

Participants were categorized into 4 mutually exclusive medication user groups within each medication category based on their prescription redemption patterns: nonuse, prior use, new use, and new persistent use (Fig. 1). Prior users were individuals who had redeemed at least one prescription for any of the 3 medication categories during the 12 months preceding the MVC, regardless of postcollision medication redemption. New users were individuals who had not redeemed any of the 3 medication categories during the year before the collision but had redeemed at least one prescription within 0 to 3 months after the collision. New persistent users were those who met the definition of new use and redeemed at least one additional prescription between 3 and 12 months postcollision. Finally, nonusers were individuals who redeemed no prescriptions for any of the 3 medication categories. To account for the limited sample size and low frequency of redeemed prescriptions, data were aggregated into 3 four-month periods before and after the MVC and medications were grouped into 3 therapeutic categories for analysis (Table 1).

**Figure 1.**
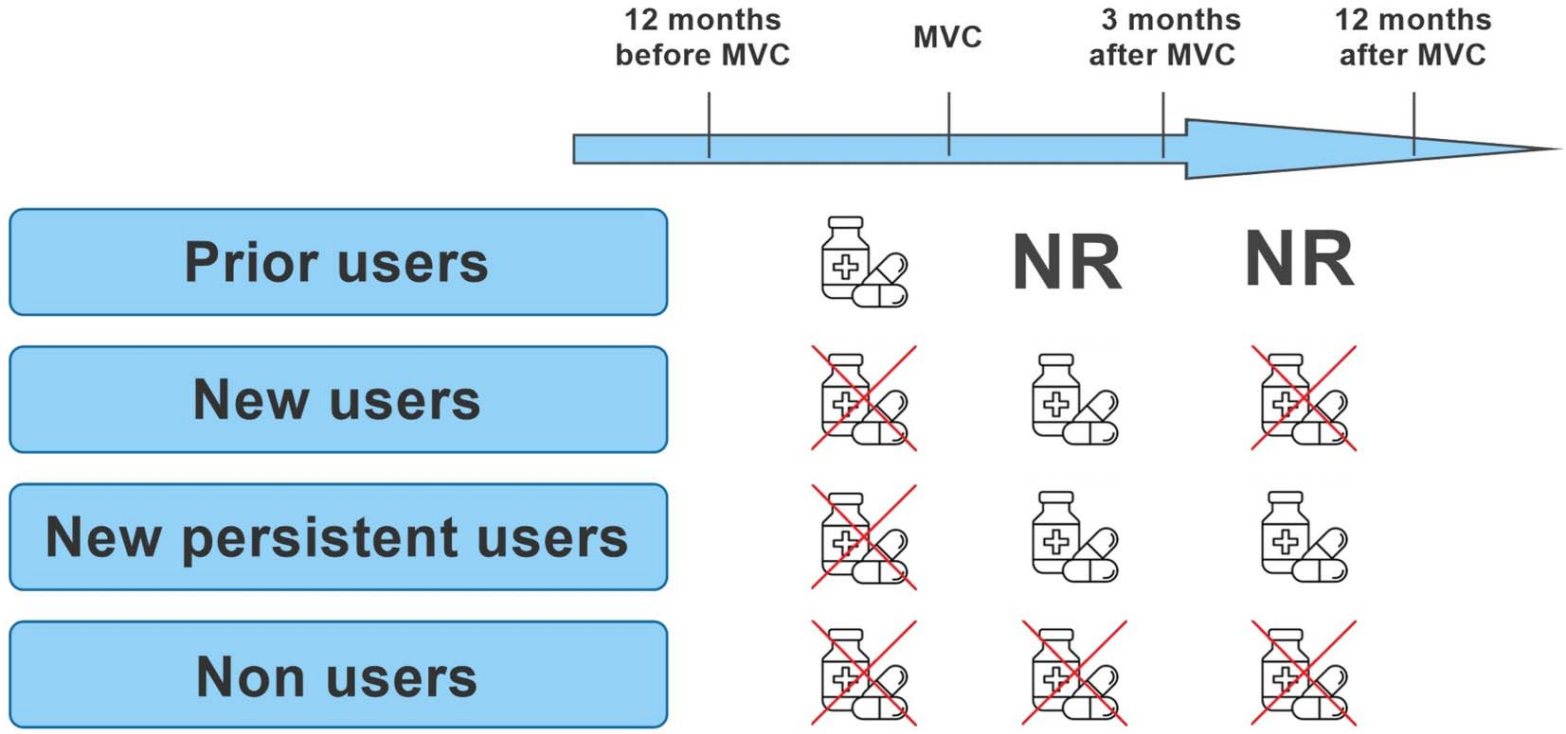
Definition of 4 mutually exclusive medication user groups based on participants' prescription redemption before and after a motor vehicle collision (MVC). NR, not relevant, ie, medication use during this period was irrelevant for the classification.

### 2.4. Statistical analysis

Data were analyzed using Microsoft Excel and Jamovi statistical software (version 2.3.28; GAMLj module). In addition to descriptive statistics, exploratory comparisons of VAS and NDI scores between sexes were conducted using independent samples *t*-tests, and simple linear regression was used to describe the relationship between age and both VAS and NDI scores. These analyses were performed as supplementary descriptive assessments to contextualize the sample. Redeemed medication was analyzed as log-transformed DDDs (logDDDs) per timepoint. We fitted a prespecified core linear mixed-effects model with a random intercept for participant and fixed effects for timepoint (six four-month periods: three before and three after the MVC), drug category, and their interaction, adjusted for sex, age, and either subacute NDI or VAS scores (entered in alternative specifications to limit collinearity). Socioeconomic status (SES), early symptom presentation, and NDI*drug category and VAS*drug category interactions were examined only in sensitivity analyses, with education used as a single SES proxy. In addition, a “core” mixed-effects logistic regression model was fitted to assess changes in proportions of individuals redeeming medication, using a binary variable as the dependent variable. The proportions of new, new persistent, and prior users were described and further analyzed using binomial logistic regression with the same covariates. Only prespecified predictors were included to limit overfitting given the modest sample size. Medication categories were analyzed independently. Consequently, participants could contribute to more than one medication category if they redeemed prescriptions from multiple drug classes during the study period. Similarly, each medication use trajectory (prior use, new use, and new persistent use) was analyzed as a separate outcome.

## 3. Results

A total of 167 individuals consented to participate in the study and met the inclusion criteria (Fig. 2). Table 2 summarizes participants' demographics and accident-related information. Women generally reported higher pain intensity on the VAS scale (6.48 SD 2.20 vs 6.03 SD 2.29, *P* = 0.019) and increased disability through the NDI questionnaire (41.9 SD 19.4 vs 33.1 SD 17.8. *P* < 0.001) compared with men and age did not have statistically significant effect on either pain intensity (*P* = 0.681) or neck disability (*P* = 0.478).

**Figure 2.**
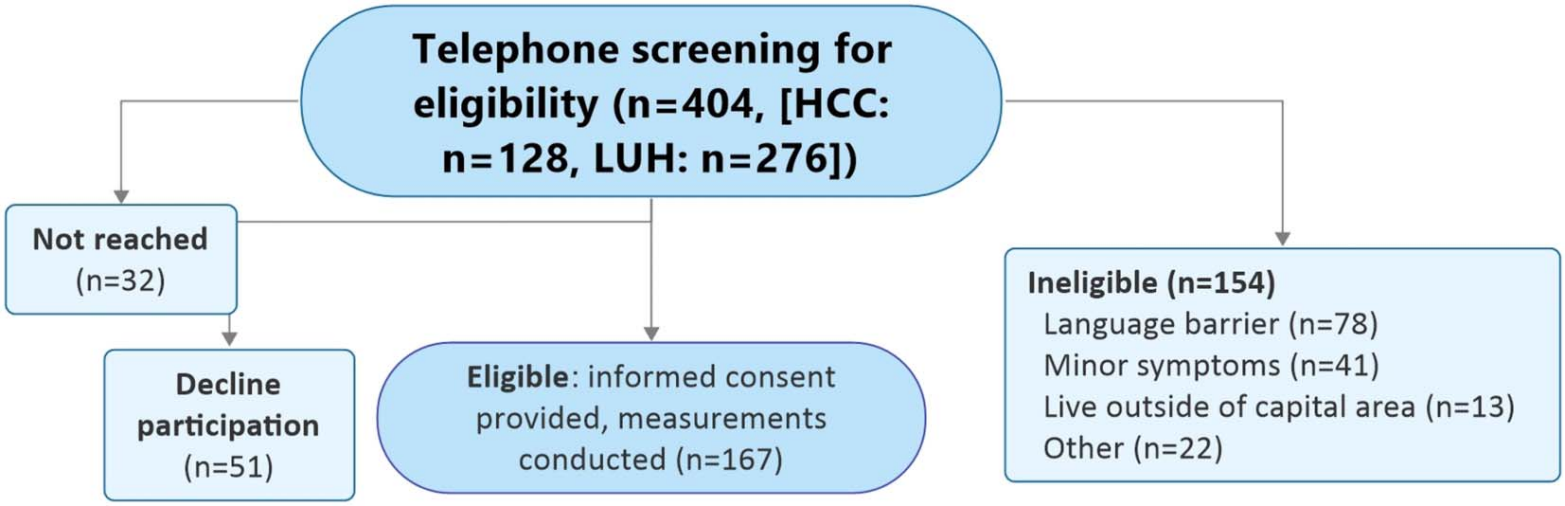
Flow diagram of participant recruitment and eligibility screening. HCC, Health Care Centers of the Capital Region; LUH, Landspitali University Hospital.

**Table 2:** Demographics and accident-related information of all participants (n = 167).

Characteristics	
Age, mean (SD) [range]	38 (13.2) [18–69]
Gender, % females	55.7
Occupation	% (n) [no answer = 6]
Worker	71.4 (115)
Contractor	3.7 (6)
Home	2.5 (4)
Student	6.8 (11)
Unemployed	2.5 (4)
Retired	2.5 (4)
Other	10.6 (17)
Work status	% (n) [no answer = 6]
Full time	58.4 (94)
Not working due to WAD	18.0 (29)
Reduced hours due to WAD	23.6 (38)
Monthly salary (1000 ISK)	% (n) [no answer = 28]
0–150	7.2 (10)
150–300	17.3 (24)
300–500	22.3 (31)
500–700	23.0 (32)
700–1000	23.7 (33)
>1000	6.5 (9)
Education	% (n) [no answer = 12]
Elementary	18.7 (29)
College/upper secondary	41.9 (65)
University degree	21.9 (34)
Postgraduate	17.4 (27)
PhD	0 (0)
Accident-related information	% (n) [no answer = n]
Position in car (driver vs passenger)	84.6 (137) [5]
Head trauma (yes vs no)	42.7 (70) [3]
Headache (yes vs no)	77.8 (126) [5]
Dizziness (yes vs no)	49.4 (80) [5]
Neck pain onset (>24 vs < 24 h)	13.4 (22) [3]

WAD, Whiplash-Associated Disorder.

In the year before the injury, 77 participants (46%) had redeemed at least one prescription from any of the 3 medication categories. In the year after the injury, a total of 126 participants (75%) had redeemed at least one prescription. Table 3 summarizes the total DDDs and number of participants redeeming medication for each category before and after the MVC while Figure 3 displays the mean DDDs and number of participants per timepoint (each of the 3 four-month periods year before and after the MVC) for each of the 3 medication categories.

**Table 3:** Total defined daily doses (DDDs), number and proportion of participants who redeemed any medication and DDDs per person per year (DDDs/pers/yr) year before and after a motor vehicle collision, by medicine category.

	12 mo before MVC		12 mo after MVC	
	Participants, n (%)	DDDs/pers/yr, median (IQR)*	Participants, n (%)	DDDs/pers/yr, median (IQR)
OTC† analgesics	31 (19)	5.1 (3.0–16.7)	58 (35)	15 (5.0–44.6)
Opioids	57 (34)	6.7 (5.0–16.7)	86 (51)	10.4 (5.0–25.0)
Sedatives/hypnotics	21 (13)	5.0 (3.0–86.0)	28 (17)	6.0 (3.0–98.1)
Total	77 (46)	6.0 (5.0–16.7)	126 (75)	13.7 (5.0–33.3)

* DDDs/pers/yr presented as median (IQR) among participants who redeemed the medication.

† OTC: Analgesics available over the counter in pharmacies.

**Figure 3.**
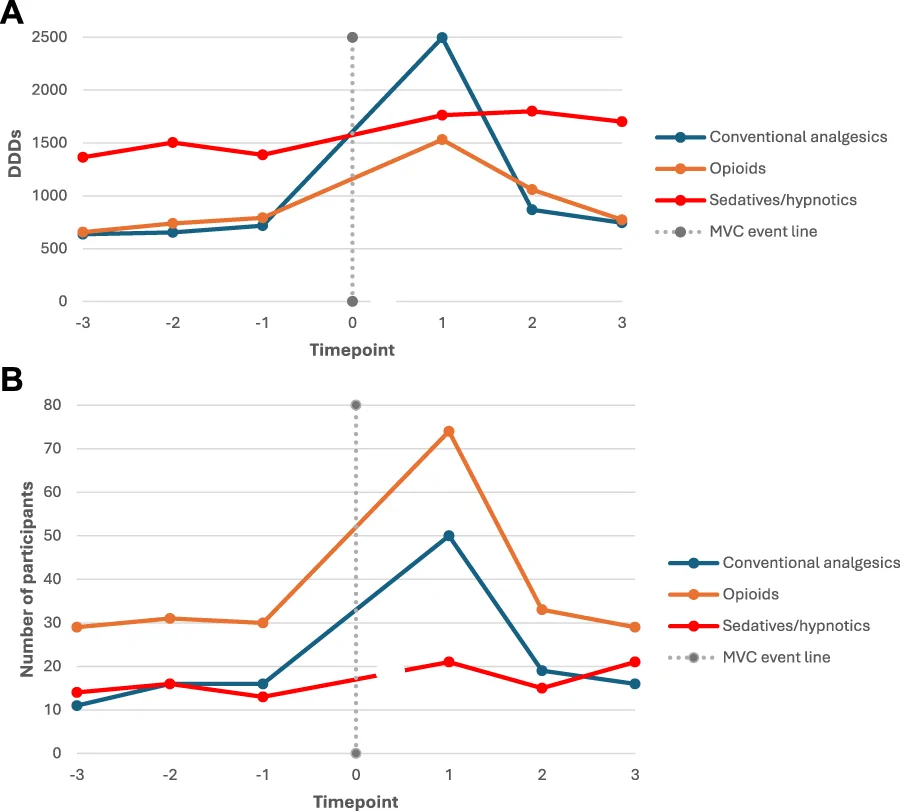
Defined daily doses (DDDs) redeemed (A) and number of participants who redeemed medication (B) across 6 timepoints, representing 3 consecutive 4-month periods before (timepoints −3 to −1) and after (timepoints 1–3) a motor vehicle collision, shown by medication category (total cohort).

A linear mixed-effects model examining logDDDs revealed expected significant main effects of drug category (F[2, 2686] = 15.31, *P* < 0.001), time point (F[5, 2686] = 18.80, *P* < 0.001), age (F[1, 155] = 20.28, *P* < 0.001), NDI (F[1, 155] = 41.40, *P* < 0.001), and VAS (F[1, 154] = 9.04, *P* = 0.003) while sex did not influence DDDs. Importantly, a significant drug category × time point interaction (F[10, 2686] = 3.46, *P* < 0.001) indicated that temporal changes in medication use differed across categories, with the greatest increase occurring in timepoint 1 (the first postinjury period; Fig. 3A) for categories 1 and 2. Post hoc pairwise comparisons (Holm-adjusted) confirmed that use in categories 1 and 2 increased significantly from timepoint −1 to timepoint 1 (*P* < 0.001), while no significant differences were observed between timepoints −1 and timepoints 2 or 3, respectively. No significant changes across timepoints were detected for category 3. A mixed-effects logistic regression model examining the probability of redeeming any medication at each timepoint showed a similar pattern with a significant interaction between drug category and timepoint (χ^2^[10] = 20.35, *P* = 0.026), with post hoc analysis revealing that the likelihood of redeeming increased significantly for OTC analgesics (*P* < 0.001) and opioids (*P* < 0.001) after the injury (between timepoints 1 and 2; Fig. 3B) but not for hypnotics. The latter model also revealed main effects of drug category (χ^2^[2] = 77.98, *P* < 0.001), timepoint (χ^2^[5] = 70.63, *P* < 0.001), age (χ^2^[1] = 26.29, *P* < 0.001), NDI (χ^2^[1] = 55.41, *P* < 0.001), and VAS (χ^2^[1] = 14.97, *P* < 0.001).

As illustrated in Figure 4, medication use (logDDDs) increased with higher NDI scores across all drug categories (F[2, 2684] = 22.33, *P* < 0.001; Fig. 4A), with the steepest slope observed in categories 2 and 3. A weaker positive association was observed between pain scores and medication use (F[2, 2667] = 13.56, *P* < 0.001; Fig. 4B). Sensitivity analysis supported the inclusion of these interaction terms, as model fit indices (Akaike information criterion (AIC), log-likelihood, and marginal R^2^) indicated improved model explanatory performance compared with the simpler model. However, including education as a SES covariate on the one hand and early symptom presentation as an indicator of initial symptom burden, on the other, did not meaningfully change the results. Both NDI and pain remained significant predictors of logDDD, while neither education nor initial symptom burden showed independent effects. Model fit indices (AIC/Bayesian information criterion (BIC)) improved slightly with their inclusion, indicating marginally stronger overall fit but no substantive alterations in parameter estimates.

**Figure 4.**
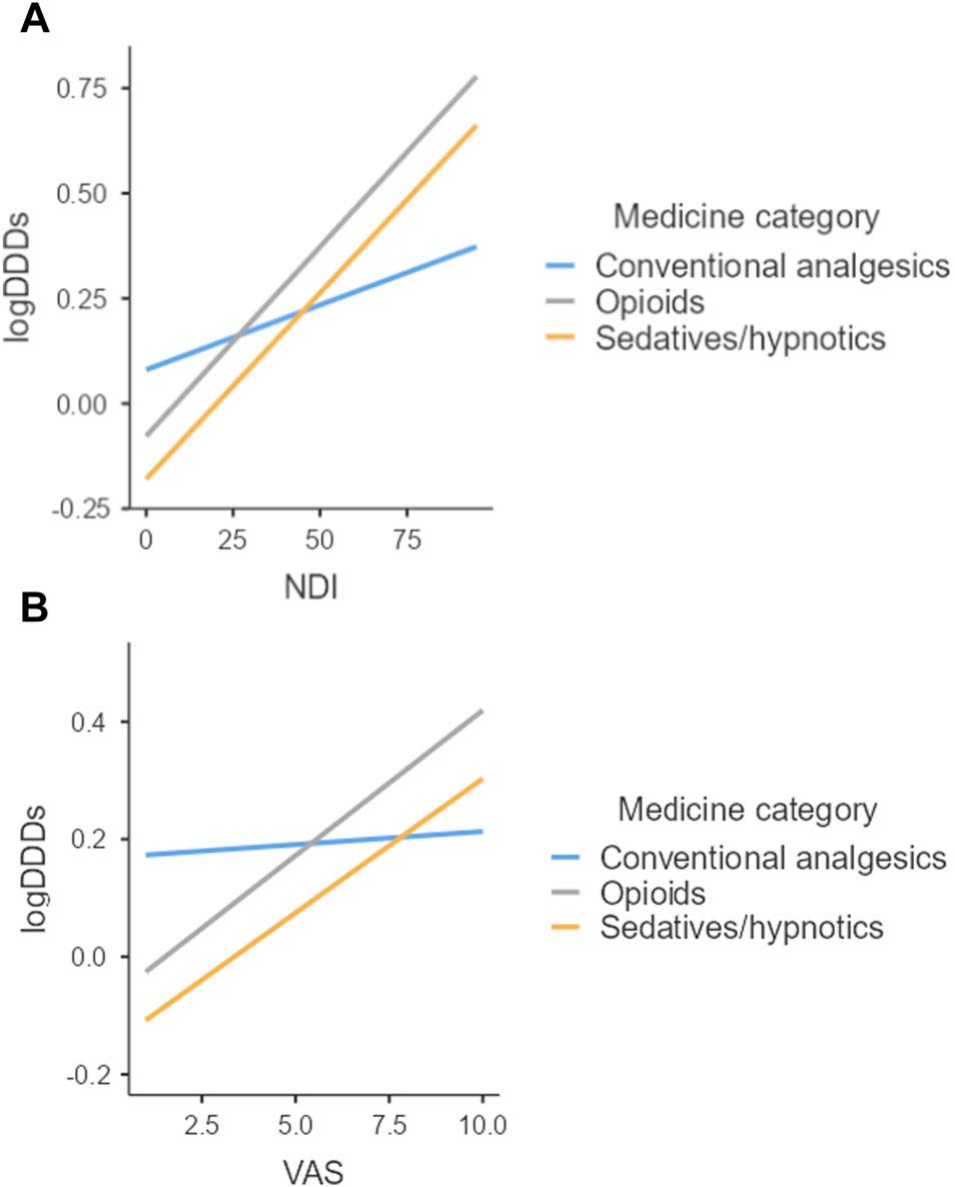
Effects of (A) disability (measured by the Neck Disability Index [NDI]) and (B) pain (measured by the Visual Analog Scale [VAS]) on log-transformed defined daily doses (logDDDs) across medication categories.

Table 4 summarizes the distribution of prior, new, and new persistent use by medication category. Note that medication categories were analyzed independently, and the same participant could belong to multiple medication categories. Results reveal that of new users, 12 OTC users (26%), 14 opioid users (26%), and 6 sedatives/hypnotics users (43%) became new persistent users.

**Table 4:** Distribution of prior, new, and new persistent use by medication category and concomitant opioid-sedative/hypnotic use.

	Prior user n (%)	New use n (%)	New persistent use n (%)	Non-use n (%)
OTC* analgesics	31 (19)	35 (21)	12 (7)	89 (53)
Opioids	57 (34)	40 (24)	14 (8)	56 (34)
Sedatives/hypnotics	21 (13)	8 (5)	6 (4)	132 (79)
Concomitant use	10 (6)	7 (4)	2 (1)	147 (88)

* OTC: analgesics available over the counter in pharmacies.

Younger participants (odds ratio [OR] = 0.98, 95% confidence interval [CI] [0.96, 1.00], *P* = 0.021) and women (OR = 1.56, 95% CI [1.01, 2.40], *P* = 0.044) were more likely to become new users of OTC analgesics compared with older or men, whereas for new opioid use, sex was the only significant predictor, indicating that women had higher odds of initiating opioid use than men (OR = 1.93, 95% CI [1.28, 2.91], *P* = 0.002). For hypnotics/sedatives (OR = 1.02, 95% CI [1.00–1.05], *P* = 0.043) and concomitant use (OR = 1.03, 95% CI [1.01–1.06], *P* = 0.011), higher disability scores were associated with increased odds of initiating use. However, these 2 models did not reach significance overall (*P* = 0.089 and *P* = 0.051, respectively).

Younger age was associated with a higher likelihood of new persistent use (OR = 0.97, 95% CI [0.95–1.00], *P* = 0.042) in OTC analgesics; however, the overall model was not significant (*P* = 0.135). For opioids, women were more likely than men to continue opioid use (OR = 2.67, 95% CI [1.45–4.91], *P* = 0.002), and higher NDI scores (OR = 1.02, 95% CI [1.01–1.04], *P* = 0.005) and higher VAS (OR = 1.21, 95% CI [1.04–1.41], *P* = 0.016) were associated with greater odds of persistence. For sedatives and hypnotics, younger participants were more likely to continue use than older (OR = 0.95, 95% CI [0.91–1.00], *P* = 0.035) and higher NDI was associated with new persistent use (OR = 1.03, 95% CI [1.00–1.06], *P* = 0.022). Finally, for concomitant use, both older age (OR = 1.07, 95% CI [1.01–1.14], *P* = 0.020) and higher NDI (OR = 1.08, 95% CI [1.03–1.14], *P* = 0.003) were associated with continued use.

Older participants were more likely to be classified as prior users of OTC analgesics (OR = 1.05, 95% CI [1.03–1.07], *P* < 0.001), opioids (OR = 1.04, 95% CI [1.03–1.06], *P* < 0.001), and sedatives/hypnotics (OR = 1.07, 95% CI [1.05–1.10], *P* < 0.001). Participants with higher NDI scores were more likely to be prior users of OTC analgesics (OR = 1.01, 95% CI [1.03–1.03], *P* = 0.014), opioids (OR = 1.03, 95% CI [1.03–1.04], *P* < 0.001), and sedatives/hypnotics (OR = 1.05, 95% CI [1.04–1.07], *P* < 0.001). Finally, men were less likely to be prior sedatives/hypnotics users than women (OR = 0.26, 95% CI [0.13–0.52], *P* < 0.001).

Sensitivity analyses including education as a proxy for SES or early symptom presentation did not meaningfully improve model fit (ΔAIC < 15) and neither variable demonstrated a significant independent effect. Moreover, their inclusion did not alter the direction or significance of parameter estimates for any of the models when exploring distribution between new use and new persistent use. All models were overall significant (*P* < 0.005) unless otherwise stated.

## 4. Discussions

This study provides novel longitudinal evidence on medication-redemption patterns before and after whiplash injury. To the best of our knowledge, it is the first study to quantify pre- and postwhiplash injury changes in redeemed medication across multiple drug categories while simultaneously examining associations with validated measures of pain and disability. While observing a significant postinjury increase (DDDs and the proportion of individuals redeeming medication) in the OTC analgesics and opioids groups, we also noted that a large proportion of individuals initiating use in opioid or sedatives/hypnotics medicine categories became new persistent users. Approximately 1 of 4 (26%) and close to half (43%), respectively, progressed to persistent use. These findings are supported by prior research^17,20,49^ and indicate that initiation itself may represent a critical inflection point at which some individuals transition toward persistent use. We further found that higher disability (according to NDI) and pain intensity (according to VAS) in the subacute phase of WAD were consistently associated with greater medication use after injury, and women were substantially more likely than men to become both new and new persistent users of OTC analgesics and opioids, aligning with the previously stated primary hypothesis. Together, these findings highlight distinct and clinically relevant trajectories of medication use after MVC-related whiplash injuries and underscore the importance of early symptom burden in influencing postinjury pharmacological behavior.

Very few studies have examined medication use both before and after an MVC.^11,49^ In older populations, reported rates of new use varied between the 2 studies that we found with one reporting 8.4% for opioids and 3.3% for sedative/hypnotics in one study,^49^ and the other reporting 27.4% for opioids and 4.4% for sedative/hypnotics in another.^11^ These findings align with our observation of a sharp early postinjury increase, particularly for opioids, followed by a more modest pattern over time. However, progression to persistent use was relatively uncommon in previous studies, with only 2% of participants becoming persistent opioid users and 1.8% becoming persistent sedatives/hypnotics users.^11^

Although we did not observe a statistically significant increase in sedative/hypnotic medication redemption during the first 4 months postinjury, the pattern nevertheless trended upward. Duchesne et al.^11^ identified a small subgroup of *delayed chronic* sedative/hypnotic users (initiation >6 months postincident), representing approximately 1% of their sample. A comparable pattern may be tentatively observed in our data. As illustrated in Figure 3, the number of sedative/hypnotic users increased again between timepoints 2 and 3 postinjury. However, our follow-up period was too short to confirm whether this indicates true delayed persistent use, and importantly, this increase was not statistically significant. Any interpretation of delayed-onset sedative/hypnotic use in our cohort should therefore be made with caution.

Socioeconomic status (ie, education level) was not identified as a significant predictor of new or new persistent use for any medication category. This contrasts with findings from research on bicycle-related injuries in Sweden, where higher education was associated with reduced long-term opioid use.^48^ Previous work has similarly linked lower socioeconomic status to greater risk of chronic opioid use.^2,18^ In contrast, sex-based differences in our cohort aligned closely with earlier studies.^6,18,38^ Women were substantially more likely to initiate and persist in opioid use. This may reflect established gender differences in pain reporting and symptom burden, including greater sensitivity to noxious stimuli,^5^ higher pain intensity,^4^ and increased prevalence of post-traumatic symptoms (including after whiplash),^22,23,47^ depression,^24^ and sleep disturbance.^39^ However, gender differences in healthcare-seeking behavior and prescribing practices may also contribute, as women generally have higher healthcare utilization^44^ and may therefore have more opportunities for medication prescribing.^1,28^

Age-related trajectories were more nuanced. Younger individuals were more likely to initiate OTC analgesic use and to become persistent OTC analgesic users or sedative/hypnotic users, whereas older adults were more likely to become persistent concomitant (opioid-sedative/hypnotic) users. Evidence regarding age-related differences in pain perception remains mixed,^25,29^ and importantly, age was not associated with pain intensity (VAS) or disability (NDI) in our sample. Thus, symptom severity does not seem to explain the higher likelihood of OTC analgesic initiation among younger individuals. A more plausible explanation is that older adults were already using analgesics before injury, thereby classifying them as prior rather than new users, this is supported by our results and by other researchers.^27^ Because OTC analgesics are available without prescription, age-related patterns in their use should be interpreted with caution. The increased likelihood of persistent concomitant opioid-sedative/hypnotic use among older adults aligns with literature linking ageing to cumulative multisystem burden (eg, cardiovascular, neurological, renal, gastrointestinal, respiratory, and musculoskeletal).^29^ Such factors may indicate a more complex postinjury symptom profile and greater pharmacological vulnerability in older individuals. Importantly, prior research has shown that early prescribing behavior, such as higher initial doses, longer initial treatment duration, repeated prescription redemption, and overlapping opioid-sedative/hypnotic prescriptions, predict long-term opioid use after injury.^12^ These findings further emphasize that early prescribing decisions may represent a pivotal moment in determining long-term trajectories. Clear documentation of indication, predefined treatment duration, and structured follow-up before prescription renewal may be critical to prevent unintended progression to persistent use. In addition, interdisciplinary monitoring, including nonprescribing clinicians such as physiotherapists, may facilitate earlier identification of patients at risk of sustained pharmacological reliance.

In light of our findings, these patterns may be particularly relevant in individuals with characteristics that increased the likelihood of becoming persistent users in our cohort, namely female sex, younger or older age depending on medication class, and higher NDI and (for opioids) VAS scores. This underscores the importance of incorporating patient-specific risk factors for persistent and potentially harmful medication use into early prescribing decisions, in addition to adhering to general guideline restrictions, to mitigate the risk of transition from short-term postinjury use to long-term or high-risk use.

## 5. Strengths and limitations

A key strength of this study is its prospective design; linking collected, validated, clinical data with high-quality nationwide prescription registry data. This integration enabled objective and longitudinal quantification of both the amount (DDDs) and patterns (prior, new, new persistent, and nonuse) of redeemed medication during the 12 months before and after an MVC, while minimizing recall bias. In addition, the prospective, nested cohort design with clearly defined WAD grade I–II cases and a preregistered study protocol reduced selection and reporting bias. The parallel analysis of categorical user trajectories and log-transformed DDDs captured complementary aspects of medication use, strengthening interpretability beyond new-use estimates alone.

Among the limitations, the small sample size required aggregation into four-month periods, reducing temporal resolution, particularly for short-lived changes early after injury. Although prespecified predictors, limited model complexity, and mixed-effects models with random intercepts were used to help minimize instability and overfitting, new-use analyses were conditional on preinjury medication status, potentially underestimating age-related effects among older adults with prevalent baseline use. Registry data reflect redeemed prescriptions rather than actual consumption and do not capture in-hospital treatment or nonprescription purchases, likely leading to underestimation of conventional analgesic use. Furthermore, prescribing indication was not available in the registry data. Medications may therefore have been redeemed for conditions unrelated to the whiplash injury, introducing potential misclassification of injury-related medication. In addition, information on additional traumatic injuries was not consistently available across the cohort. Consequently, we could not account for the potential influence of concomitant injuries on medication redemption after the collision. While nationwide prescription data enhance the completeness of medication outcome assessment, the cohort itself reflects care-seeking individuals from a single healthcare region. Caution is therefore warranted when extrapolating these findings to less severe WAD cases, non–care-seeking populations, or healthcare systems with different prescribing practices.

The present analyses focused on whether early postinjury clinical characteristics predicted subsequent medication use trajectories. Consequently, we did not examine whether persistent medication use was associated with pain or disability at 12 months. Future studies should investigate the relationship between long-term medication use and clinical outcomes.

## 6. Conclusions

In this population-based cohort, we found clear increases in medication redemption after whiplash injury. Notably, 26% of new opioid users and 43% of new sedatives/hypnotics users progressed to persistent use. Female sex, age, and higher pain and disability scores emerged as key predictors of these trajectories. Given that early prescribing is a strong determinant of long-term opioid use, these factors may help identify individuals at particular risk for sustained pharmacological dependence. Our results underscore the importance of cautious, time-limited medication use and early, guideline-concordant nonpharmacological management in the care of patients with whiplash injuries.

## Disclosures

The authors have no conflict of interest to declare.
